# Gene Mutations of the Three Ectodysplasin Pathway Key Players (*EDA*, *EDAR*, and *EDARADD*) Account for More than 60% of Egyptian Ectodermal Dysplasia: A Report of Seven Novel Mutations

**DOI:** 10.3390/genes12091389

**Published:** 2021-09-08

**Authors:** Hoda A. Ahmed, Ghada Y. El-Kamah, Eman Rabie, Mostafa I. Mostafa, Maha R. Abouzaid, Nehal F. Hassib, Mennat I. Mehrez, Mohamed A. Abdel-Kader, Yasmine H. Mohsen, Suher K. Zada, Khalda S. Amr, Inas S. M. Sayed

**Affiliations:** 1 Medical Molecular Genetics Department, Human Genetics & Genome Research Division (HGGR), National Research Centre (NRC), Cairo 12622, Egypt; hoda_radwan80@yahoo.com; 2 Clinical Genetics Department, Human Genetics & Genome Research Division (HGGR), National Research Centre (NRC), Cairo 12622, Egypt; gg.kamah@nrc.sci.eg; 3 Biology Department, School of Sciences and Engineering, The American University in Cairo (AUC), Cairo 11835, Egypt; suzada@aucegypt.edu; 4 Orodental Genetics Department, Human Genetics & Genome Research Division (HGGR), National Research Centre (NRC), Cairo 12622, Egypt; mostafanrc@yahoo.com (M.I.M.); ooah119@gmail.com (M.R.A.); nounih@hotmail.com (N.F.H.); mehrezmi@outlook.com (M.I.M.); mohamedabdelkader1979@gmail.com (M.A.A.-K.); yasminehm@hotmail.com (Y.H.M.); inas_sayed@hotmail.com (I.S.M.S.)

**Keywords:** ectodysplasin A, ectodermal eysplasia, *EDA*, *EDAR*, *EDARADD*, *WNT10A*, hypohidrotic ectodermal eysplasia, oligodontia, hypodontia

## Abstract

Ectodermal dysplasia (ED) is a diverse group of genetic disorders caused by congenital defects of two or more ectodermal-derived body structures, namely, hair, teeth, nails, and some glands, e.g., sweat glands. Molecular pathogenesis of ED involves mutations of genes encoding key proteins of major developmental pathways, including ectodysplasin (EDA) and wingless-type (WNT) pathways. The most common ED phenotype is hypohidrotic/anhidrotic ectodermal dysplasia (HED) featuring hypotrichosis, hypohidrosis/anhidrosis, and hypodontia. Molecular diagnosis is fundamental for disease management and emerging treatments. We used targeted next generation sequencing to study *EDA*, *EDAR*, *EDARADD*, and *WNT10A* genes in 45 Egyptian ED patients with or without hypohidrosis. We present genotype and phenotype data of 28 molecularly-characterized patients demonstrating genetic heterogeneity, variable expressivity, and intrafamilial phenotypic variability. Thirteen mutations were reported, including four novel *EDA* mutations, two novel *EDARADD*, and one novel *EDAR* mutations. Identified mutations congregated in exons encoding key functional domains. *EDA* is the most common gene contributing to 85% of the identified Egyptian ED genetic spectrum, followed by *EDARADD* (10%) and *EDAR* (5%). Our cohort represents the first and largest cohort from North Africa where more than 60% of ED patients were identified emphasizing the need for exome sequencing to explore unidentified cases.

## 1. Introduction

Ectodermal dysplasia (ED) is a heterogenous group of genetic disorders caused by congenital defects affecting the development and/or homeostasis of two or more ectodermal-derived body structures, namely, hair, teeth, nails, and certain glandular structures, e.g., sweat, salivary, and sebaceous glands [1]. Seventy-seven genes and nine chromosomal regions are linked to 75 types of EDs [2]. The most common ED phenotype is hypohidrotic/anhidrotic ectodermal dysplasia (HED) which represents about 50% of ED [3,4]. The HED phenotype comprises three cardinal features: hypotrichosis (dry, thin, and sparse hair), hypohidrosis/anhidrosis (few to no sweat glands), and dental anomalies, including congenital absence of several deciduous and permanent teeth, abnormally shaped teeth, and microdontia. Other features might include frontal bossing, periorbital hyperpigmentation, saddle nose, and prominent lips [5,6,7]. Other ED phenotypes include the impairment of at least two ectodermal derivatives, e.g., ED of hair/nail type, ED of hair/tooth/nail type, and ED without hypohidrosis. These different ED phenotypes are caused by monogenic defects in genes encoding key player proteins of multiple major developmental pathways, e.g., ectodysplasin (EDA), wingless-type (WNT), and tumor protein p63 (TP63) pathways [6,8,9]. Some phenotypic features were frequently linked to a definite pathway, e.g., cleft lip/palate and hand/foot malformations are linked to TP63 pathway [9,10].

Different modes of inheritance have been described for different ED phenotypes, e.g., HED can be inherited as autosomal dominant, autosomal recessive, or the most common X-linked form (XLHED). Owing to clinical heterogeneity, prevalence of ED as a whole is seldom described. Prevalence of HED is 1–9 per 100,000 births and is expected to be higher in populations with high consanguinity rates [11,12].

In HED, features range from mild to severe, and most patients have a normal life span given early diagnosis and symptomatic management. Nonetheless, quality of life is affected by hypodontia, dental malformations, and their consequent growth problems, given the high cost of dental management [13]. Furthermore, reduced/absent sweating increases the risk for hyperthermia, and dryness of airways causes recurrent infections; both lead to life-threating complications [14]. Identifying the exact molecular defect underlying HED condition is vital for early diagnosis and the application of emerging and future therapies [15,16].

The involvement of EDA and WNT signaling pathways have been described in HED as well as other ED phenotypes [1]. The EDA signaling pathway plays a pivotal role in the initiation and progression of ectodermal morphogenesis and organogenesis during embryogenesis [17]. The *EDA* gene (chromosome chrXq12–q13.1) encodes a trimeric transmembrane protein ligand (Eda), which is cleaved by furin to give a soluble and active secreted form that is capable of binding tumor necrosis factor (TNF) transmembrane receptor called ectodysplasin receptor (Edar, encoded by *EDAR*, located on chr2q11–q13). Edar is attached to an adaptor molecule called EDAR associated death domain (Edaradd, encoded by *EDARADD*, located on chr1q42–q43). Binding of Eda to Edar activates Edaradd to recruit further cytoplasmic adaptor molecules, including: Traf6 (TNF receptor associated factor), Tab2 (Tak1 binding protein), and Tak1 (transforming growth factor β activated kinase). Tak1 activates the inhibitor of nuclear factor kappa B kinase complex (IKK). IKK phosphorylates the inhibitor of nuclear factor kappa B (I-NFκB), resulting in its degradation and the release of NFκB, which enters the nucleus to activate target genes involved in ectodermal organogenesis [17,18]. The interaction between Eda and the Wnt/β-catenin pathway elucidates the involvement of Wnt10a (wingless-type 10A) with the HED phenotype. Edar expression is induced by the Wnt/β catenin pathway while Eda pathway maintains the expression of the ligand Wnt10a during early embryogenesis [19].

Mutations in the *EDA* gene are responsible for XLHED, whereas mutations in *EDAR*, *EDARADD*, and *WNT10A* genes are responsible for ED of autosomal transmission (non XLHED). The four aforementioned genes contribute to approximately 90% of HED cases in Caucasians, whilst 10% of cases remain unidentified [6]. Ethnicity may play a role in the contribution of these four genes in HED spectra [20,21,22]. ED represents 10% of pediatric genetic skin disorders in Egypt; however, only two XLHED families were described to have *EDA* mutations [23,24].

Our study aims at exploration of the molecular and mutation spectra in a cohort of 45 Egyptian ED patients with or without hypohidrosis, which is the first and largest cohort from North Africa. We employed targeted next generation sequencing (NGS) to screen *EDA*, *EDAR*, *EDARADD*, and *WNT10A* mutations and investigated the genotypic and phenotypic heterogeneities of molecularly characterized patients.

## 2. Patients and Methods

### 2.1. Ethical Considerations

Patients were recruited to the Orodental and Genodermatoses outpatient clinics of the Medical Research Excellence Centre of the National Research Centre (NRC) of Egypt. Written informed consent was obtained from all participants and/or legal guardians. This study was approved by the Medical Ethics Committee of NRC and the institutional review board of the American University in Cairo (AUC) according to the Helsinki declaration.

### 2.2. Clinical Evaluation

Our study included 45 Egyptian ED patients descending from 35 unrelated families. Patients were selected based on the impairment of at least two ectodermal derivatives. Exclusion criteria included patients with non-syndromic traits, i.e., where one ectodermal derivative only was impaired, and patients having syndromic ED features, e.g., cleft lip/palate, hand/foot malformations, and hearing loss. Patients were subjected to full medical history, including sex, age, chief complaint, progression of symptoms, the presence of any birth or pregnancy complications, and history of present illness. Pedigree analysis for at least three generations with emphasis on consanguinity and family members having similar or different genetic abnormalities was done. Patients were evaluated by a multidisciplinary team, including a clinical geneticist, a dentist, a dermatologist, and an otolaryngologist. General clinical examination focused on the skin and other ectodermal elements, including the presence of sparse/brittle hair, dry skin, dystrophic nails, and history of hyperthermia. Patients and parents underwent a thorough oral and dental examination. Dentition was assessed by panoramic radiographs in cooperative patients older than five years. Hypodontia was defined as agenesis of less than six teeth while oligodontia is the agenesis of six teeth or more. Patients younger than three years with delayed eruption of teeth in the presence of conical peg shaped teeth and/or in the presence of other ectodermal manifestations were also included in the study.

### 2.3. Molecular Investigation

#### 2.3.1. Genomic DNA Extraction and Targeted NGS

DNA was extracted from peripheral blood samples of all patients, parents, and available family members using standard salting out procedure. DNA samples of patients were quantified using fluorometric Denovix Qubit™ dsDNA BR Assay Kit (ThermoFisher, Waltham, MA, USA) and diluted to 6–20 pM final loading concentration, as per custom panel recommendation. Ampliseq On Demand NGS custom panel from Illumina (ThermoFisher, Waltham, MA, USA) was designed, and the targeted genomic regions included the exons, around 100 bp of flanking intronic sequences, and the 5′and 3′ un-translated regions of *EDA*, *EDAR*, *EDARADD*, and *WNT10A* genes. The average coverage depth of the entire panel was 100×, and ~85–100% of targeted bases were covered. The NGS library was prepared using Ampliseq Custom Amplicon kit (ThermoFisher, Waltham, MA, USA) according to manufacturer’s reference guide (Illumina #1000000036408). Briefly, 100 ng of genomic DNA in 7.5 μL water were hybridized with an oligo pool. Then, unbound oligos were removed, and extension-ligation of bound oligos was followed by PCR amplification. PCR products were cleaned. Prior to sequencing, libraries were normalized with the normalization process of the Ampliseq Custom Amplicon kit. Libraries were paired-end sequenced with 2 × 151 basepairs cycles on a MiSeq device (Illumina, San Diego, CA, USA).

#### 2.3.2. Data Processing and Bioinformatic Analysis

The generated sequence data from enriched libraries were analyzed by MiSeq reporter software. FASTQ files were generated and Burrows Wheeler Aligner (BWA) was used to align the reads against the GRCh37/hg19 reference genome to create BAM files. Genome Analysis Toolkit (GATK) was used for variant analysis for the included target regions of the specified genes. Variant filtration and analysis were carried out using Illumina Variant Studio software. Variants were analyses based on satisfying sequencing depth, quality, and frequency in dbSNP [25], Genome Aggregation Database (GnomAD) [26], and 1000 Genomes Project (1000G) [27] databases. Variants were named according to Human Genome Variation Society (http://www.hgvs.org, accessed on 10 January 2021). Pathogenicity of variants was interpreted based on the recommended standards of the American College of Medical Genetics and Genomics (ACMG) [28]. For novel missense variants, eight in silico prediction algorithms were used to assess the impact of these variants on protein structure/function and evolutionary conservation. For evolutionary conservation, we used SIFT [29], PhD-SNP [30], and Mutation Assessor [31]. Polyphen-2 [32], MutationTaster2 [33], and PROVEAN [34] were used for the impact on both the protein structure/function and evolutionary conservation. SNPs&GO takes gene ontology into account while CADD contrasts annotations of fixed derived alleles in humans with simulated variants of interest [35,36]. Novel variants were deposited in ClinVar database [37].

#### 2.3.3. Segregation Analyses

Sanger sequencing was used for segregation analyses among affected siblings, parents, and available family members of molecularly characterized patients. Primers were designed using Primer3 tool (https://primer3.ut.ee/, accessed on 10 January 2021), Table 1. PCR cycling conditions started by initial denaturation at 96 °C for 5 min followed by 30 three-stage cycles. Each cycle included (1) denaturation at 96 °C for 30 s, (2) annealing at primer pair’s specific temperature for 30 s, and (3) extension at 72 °C for 30 min. A final extension at 72 °C for 5 min followed. PCR products were purified using QIAquick PCR purification kit (Qiagen, Dusseldorf, Germany). Forward and reverse DNA strands were sequenced using the Big Dye Termination kit (Applied Biosystems, Waltham, MA, USA), and analyzed on the ABI Prism 3500 Genetic Analyzer (Applied Biosystems, Waltham, MA, USA) according to manufacturer’s protocols.

## 3. Results

### 3.1. Clinical Data of 28 Molecularly Identified ED Patients

**Figure 1:**
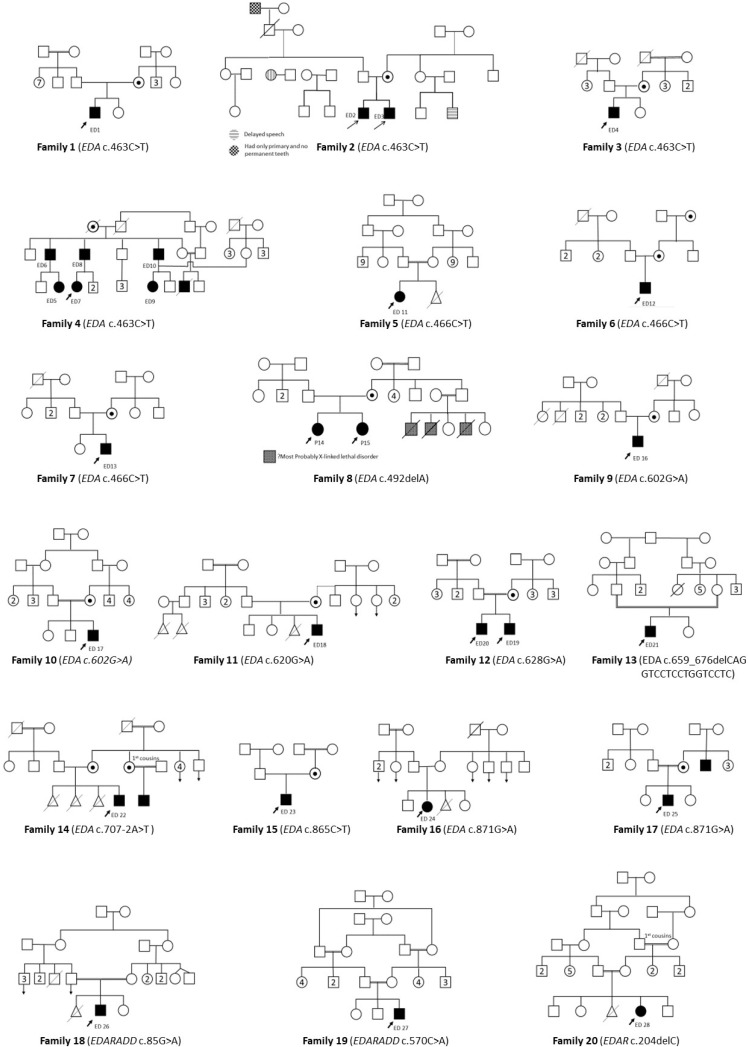
Pedigrees of molecularly identified ED patients. The figure shows 20 family pedigrees of 28 molecularly identified ED patients (ED1-ED28). Probands are denoted by black arrows, and the identified mutation is shown below each pedigree.

**Figure 2:**
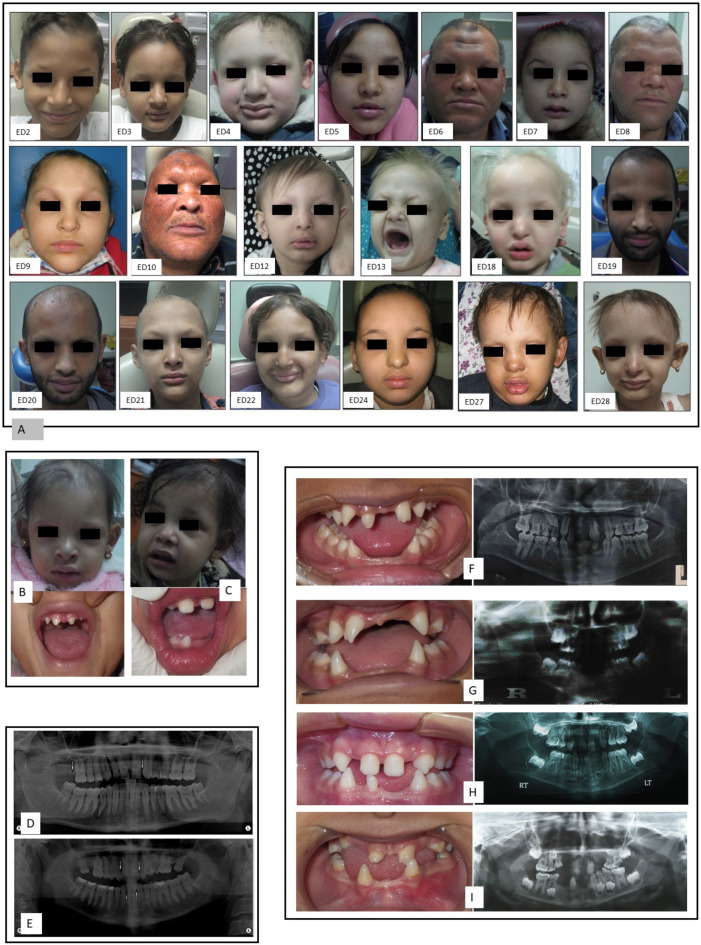
Clinical features and panoramic radiographs of ED patients. (**A**) Facial photographs of ED patients showing the typical facial features of ED, including sparse hair, thick prominent lips, and depressed nasal bridge. (**B**,**C**) Facial and intraoral photographs of ED14 and ED15, respectively, showing the difference between the severely affected ED14 in contrast to milder phenotype of her sister, ED15 of family 8. (**D**,**E**) panoramic radiographs of the father and mother of ED28, respectively, showing hypodontia (white arrows indicate sites of missing teeth), which is the only ectodermal feature in these carrier parents, in contrast to the severely affected proband, who showed hypohidrosis, sparse hair without any erupted teeth at the age of 3 years. (**F**–**I**) Intraoral photographs and panoramic radiographs of patients ED2, ED3, ED7, and ED24, respectively, showing oligodontia and peg shaped teeth.

Molecular investigation identified pathogenic variants in 28 patients (62%) descending from 20 unrelated pedigrees out of the 45 participant ED patients. The clinical data of the 28 molecularly identified patients (ED1-ED28) is summarized in Table 2. Positive parental consanguinity was detected in nine (45%) of the 20 pedigrees with characterized molecular pathology, Figure 1. Among the 28 molecularly identified patients whose ages ranged between one year and 59 years, there was a clear male preponderance (20/28, 71.4%). HED triad manifested in 19/28 (68%) of patients; in four patients (ED13, ED18, ED27, and ED28), hypohidrosis and hypotrichosis were detected, but panoramic radiographs could not be obtained owing to their young age, Figure 2. Only five patients (5/28, 17.8%) had normal sweating, i.e., hidrotic ED. Dental abnormalities were present in 24 patients (24/28, 85.7%) in form of oligodontia and/or peg shaped teeth. While almost all patients presented with dry skin and sparse hair, only six patients (6/28, 21.4%) showed dysplastic nails.

### 3.2. Genetic Spectrum of ED in Our Cohort

**Figure 3:**
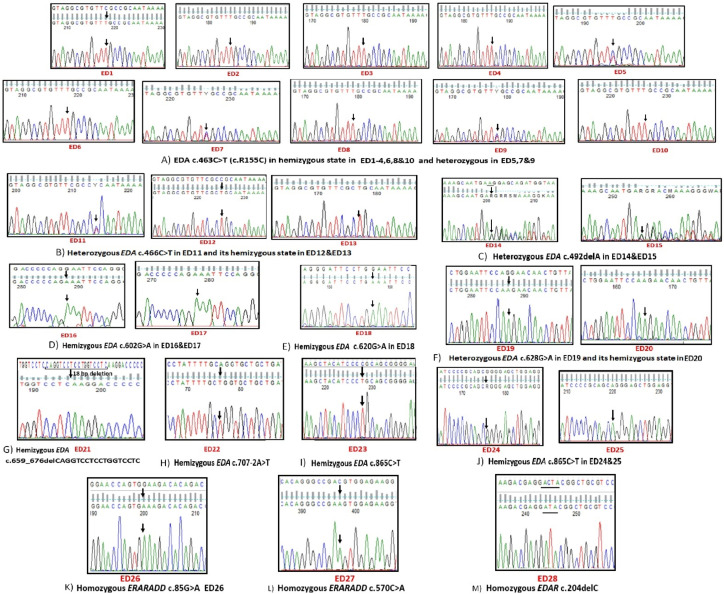
Sequencing chromatograms of 13 mutations identified in *EDA*, *EDARADD*, and *EDAR*. The figure shows 10 *EDA* (NM_001399.4) mutations (**A**–**J**), two *EDARADD* (NM_145861.2) mutations (**K**,**L**), and one *EDAR* (NM_022336.3) mutation (**M**). Above each first chromatogram of a given mutation is the wildtype sequence. Black arrows point towards base changes, and deletions are underlined. Below each chart is its corresponding patient number in red.

A total of 13 different mutations were identified and predicted to be pathogenic according to ACMG guidelines: six previously reported *EDA* mutations, four novel *EDA* mutations, two novel *EDARADD*, and one novel *EDAR* mutations, Figure 3 [28]. The 10 *EDA* (NM_001399.4) mutations were identified in 25 patients from 17 unrelated families, i.e., 85% of the studied pedigrees, two *EDARADD* (NM_145861.2) in 10% (two patients from two unrelated families), and one *EDAR* (NM_022336.3) in 5% (one patient), Table 3. All five hidrotic patients had *EDA* mutations. No *WNT10A* mutations were detected.

#### 3.2.1. *EDA* Mutations

The 25 patients harboring *EDA* mutations were 18 hemizygous males and seven heterozygous females. The most common mutation was the *EDA* missense (c.463C > T, p.R155C) mutation found in 10 patients from four unrelated pedigrees, followed by another missense *EDA* mutation (c.466C > T, p.R156C), which was identified in three patients from three unrelated pedigrees, Figure 1 and Table 3. All of the 10 identified *EDA* mutations were missense mutations except one splicing mutation (c.707-2A > T), 18 bp in-frame deletion (c.659_676delCAGGTCCTCCTGGTCCTC, p.P220_P225del), and one base pair frameshift deletion (c.492delA, p.G165Efs*115), Figure 3. All 10 *EDA* mutations were identified in exons number two, four, and seven of *EDA* gene and their flanking intronic region, Table 3.

#### 3.2.2. Seven Novel Mutations and Their in Silico Analyses

We identified seven novel mutations, which were assigned accession numbers (SCV001480041.1-SCV001480043.1, SCV001480045.1, SCV001480046.1 SCV001762294.1, and SCV001762295.1) in ClinVar database [37]. None of these variants were found in dbSNP, 1000G, gnomAD exome, and ExAC databases or in 100 chromosomes from unaffected/unrelated Egyptian subjects. Two novel frameshift mutations, *EDAR* (c.204delC, p.Y69Tfs*34) and *EDA* (c.492delA, p.G165Efs*115), were predicted to be disease causing by MutationTaster2 tool. For novel missense variants, seven in silico algorithms predicted the deleterious, damaging, or disease-causing statuses of the three novel *EDA* mutations: c.602G > A (p.G201E), c.620G > A (p.G207E), and c.628G > A (p.G210R), Table 4. The same prediction was obtained in the case of the two novel *EDARADD* variants, (c.85G > A, p.E29K) and (c.570C > A, p.D190E), in six out of seven prediction tools, Table 4. CADD scores for the five novel missense variants were greater than 20, which means that these variants are among the top 1% of deleterious variants of the human genome [36].

#### 3.2.3. Variable Expressivity of Heterozygous Carriers and De Novo Events

Among the 20 molecularly characterized families, X-linked inheritance (XL) was evident through pedigree analyses in 13 families, where mothers of respective probands showed partial manifestations, e.g., four missing teeth of the mother (hypodontia) and maternal grandmother of ED13, microdontia of upper lateral incisor of the mother of ED14 and 15. In two XL ED families, F11 and F15, heterozygous carrier mothers did not show any ED related manifestations, thus, XL inheritance was confirmed only after molecular diagnosis. In another two families, F13 and F16, ED21, and ED24 harbored two de novo *EDA* mutations, the 18 bp in-frame deletion (c.659_676delCAGGTCCTCCTGGTCCTC, p.P220_P225del) and the missense (c.871G > A, p.G291R) mutation, respectively, Figure 1. Variant segregation analysis confirmed autosomal recessive transmission of the three novel variants: missense *EDARADD* (c.85G > A, p.E29K) mutation in ED26, missense *EDARADD* (c.570C > A, p.D190E) mutation in ED27, and frameshift *EDAR* (c.204delC, p.Y69Tfs*34) mutation in ED28, Figure 3 and Table 3. Both parents of ED28 had mild hypodontia, Figure 2D,E.

## 4. Discussions

Molecular diagnosis of more than 60% of Egyptian ED patients classified as HED or hidrotic ED identified *EDA* to be the most common gene, contributing to 85% of the Egyptian genetic spectrum, followed by *EDARADD* (10%) and *EDAR* (5%). No *WNT10A* mutations were identified. The majority of our cohort were HED patients who were identified to have mutations in *EDA* (87%), *EDARADD* (8.7%), and *EDAR* (4.3%) genes, unlike hidrotic ED patients who represented 17.8% of our cohort and clustered only into *EDA* mutation spectrum. To our knowledge, our study comprises the first and largest North African cohort to investigate *EDA*, *EDAR*, *EDARADD*, and *WNT10A* genes in HED. Our genetic spectrum of HED differs from previously studied Mediterranean HED cohorts, which reported *WNT10A* mutations to account for about 16–25% of their genetic spectra and *EDARADD* mutations to be the least common [6,20,21,22,42]. Such discrepancies may be due to pan ethnic variation in populations, different cohort sizes, and/or clinical selection differences. Conclusively, the molecular defect underlying about 20–40% of HED across different studied cohorts, including ours, remains to be elucidated.

**Figure 4:**
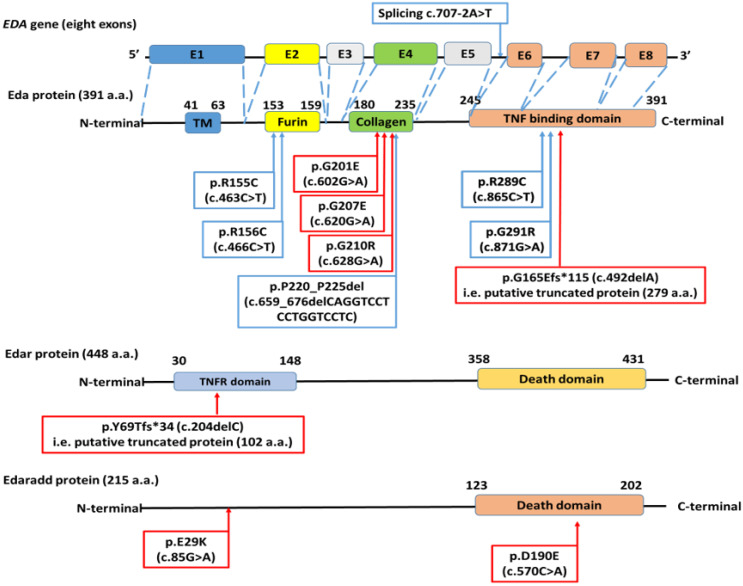
Distribution of 13 different mutation on gene and/or protein structure of *EDA*, *EDAR*, and *EDARADD*.

Our 10 identified *EDA* mutations congregated into three hotspot exons (E2, E4, and E7) encoding evolutionarily conserved and functionally relevant domains of Eda protein, Figure 4. The two most common *EDA* mutations in our cohort, (c.463C > T, p.R155C) and (c.466C > T, p.R156C), located in E2, abolish the furin cleavage site, directly impacting Eda function. Both mutations were reported to cause classical HED triad with severe skin manifestations [23,38,43,44,45,46]. The 13 patients (ED1–ED13) harboring either (c.463C > T, p.R155C) or (c.466C > T, p.R156C) had classical HED features, except for ED3, ED7, and ED9, who carried (c.463C > T, p.R155C) mutation and had hidrotic ED with milder phenotypes than other affected members of their families. To our knowledge, our study has the largest number of *EDA* patients harboring (c.463C > T, p.R155C) mutation, thus, providing evidence for intrafamilial phenotypic variability of this mutation, which makes genetic counseling, specifically concerning prenatal decision, difficult. We predict that other genetic factors might contribute to the sparing of sweat gland affection in some patients.

Seven novel (red boxes) and six previously reported (blue boxes) mutations are shown with respect to their amino acid (a.a.) position on their affected protein product. *EDA* has eight exons, which encode a 391 amino acid (a.a.) type II transmembrane protein divided into a small intracellular N terminal region, a transmembrane domain (TM), and a large C-terminal extracellular region. The domain harboring furin cleavage site (position: 153–159 a.a., encoded by exon 2 (E2)) is located at the beginning of the extracellular region, where Eda is cleaved into the secreted form to bind to Edar receptor (TNF receptor) for NF-κB pathway activation. The extracellular region harbors another two crucial functional domains; the 19 collagen-like repeats (Gly-X-Y)_19_ domain (position 180–235 a.a. encoded by E4) and a TNF homology domain (position 245-391 a.a. encoded by E6, E7, and E8) [39,47]. The *EDA* gene is shown to illustrate the congregation of identified mutations in E2, E4, and E7. Edar protein (448 a.a.) is a tumor necrosis factor receptor (TNFR) with two predicted domains: TNFR domain (30–148 a.a.) and death domain (358–431 a.a.). Edaradd protein (215 a.a.) is associated with tumor necrosis factor receptor with a death domain predicted at (123–202 a.a.) [48].

*EDA* mutations are known to cause two overlapping phenotypes: HED and non-syndromic tooth agenesis (NTA). This variation in phenotype was suggestively attributed to residual Eda receptor binding ability of some NTA-causing *EDA* mutations versus complete abolishment in the case of HED-causing *EDA* mutations [41,49]. Such a suggestion was supported by phenotypic rescue of hair and salivary glands at lower Eda doses than that required for the rescue of the teeth in canine models [50]. Adversely, the same *EDA* mutation can cause either HED or NTA; for example, we identified c.865C > T (p.R289C) in TNF binding domain of Eda in a 2-year-old hidrotic ED case with sparse hair and eruption of few peg shaped teeth (ED23); this *EDA* mutation was previously reported in isolated oligodontia cases only, thus, the involvement of other genetic factors could be suggested [41,49,51,52,53].

Other *EDA* mutations affecting the TNF domain of Eda are the previously reported c.871G > A (p.G291R) and the novel *EDA* c.492delA (p.G165Efs*115) frameshift mutation. The c.871G > A (p.G291R) mutation was exclusively reported to cause cardinal HED phenotype, which was the same case in ED24 and ED25, harboring this mutation [22,39,54]. The novel *EDA* c.492delA (p.G165Efs*115) was identified in two heterozygous sisters (ED14 and ED15); ED14 had HED with dysplastic nails, unlike ED15, who had hidrotic ED with normal nails. Both sisters had peg shaped teeth with delayed teeth eruption, suggesting tooth agenesis, but ED14 had a more severe dental phenotype. Of interest, the mother of ED14 and ED15 was a heterozygous carrier of c.871G > A (p.G291R) mutation, presenting only with microdontia of the upper lateral incisor. This variable expressivity and incomplete penetrance of heterozygous females has been reported before and supports the conclusion of the presence of other genetic factors influencing the HED phenotype [6,22].

The collagenous domain of Eda provides a critical role for protein function, particularly in the oligomerization of TNF domain of Eda into higher assemblies for further downstream Eda–Edar ligand receptor binding [47]. Three novel *EDA* mutations, c.602G > A (p.G201E) of ED16 and ED17, 620G > A (p.G207E) of ED18, and c.628G > A (p.G210R) of ED19 and ED20, affected the Eda collagenous domain, Figure 4, and were predicted as pathogenic/disease causing/deleterious by eight different in silico algorithms, Table 4. The involvement of nails was observed in the two siblings ED19 and ED20, which questions the contribution of their *EDA* c.628G > A (p.G210R) mutation;a however, a larger number of patients would be required for affirmation. The previously reported *EDA* splicing mutation (c.707-2A > T) in intron 5 was predicted to alter gene splicing via abolishment of the canonical (AG) splice acceptor site, which results in either exon skipping or the use of an alternative splice site. The *EDA* c.707-2A > T has been associated with severe HED as that observed in ED22 [40]. An 18 bp in-frame deletion (c.659_676delCAGGTCCTCCTGGTCCTC, p.P220_P225del) was identified in ED21, which deleted five a.a. and interrupted (G-X-Y)_13–15_ repeats of the Eda’s 19 collagen repeats. This deletion was suggestively attributed to unequal crossing over owing to the flanking GC-rich regions or an error in replication [39,42]. A similar proximal 18 bp deletion c.663-680delTCCTCCTGGTCCTCAAGG (p.P222_G227) was reported in an Egyptian patient with NTA. The overlapping phenotype of HED and NTA caused by the same *EDA* mutation led to the suggestion that both phenotypes represent the same disease with variable severities [23].

We showed that pedigree analyses can be misleading due to variable expressivity of heterozygous *EDA* carrier mothers, where some did not show any ED related manifestations, and the occurrence of de novo events, e.g., in ED21 and ED24. In these cases, XL inheritance was excluded, but later, XLHED was molecularly diagnosed. Consequently, *EDA* should be screened in all ED patients, whatever their prospected pedigrees. Of interest, we identified a novel frameshift *EDAR* (c.204delC, p.Y69Tfs*34) mutation in ED28 inherited in an autosomal recessive manner. Both carrier parents of ED28 had mild hypodontia, suggesting that carriers of only one affected *EDAR* allele may show mild phenotype, similar to female carriers of XL *EDA* mutations [55]. The frameshift *EDAR* (c.204delC, p.Y69Tfs*34) mutation is predicted to produce mRNA subjected to non-sense mediated decay (NMD). Even if a putative Edar truncated protein (102 a.a.) is produced, it would lack the crucial TNF receptor and death domain of wildtype Edar (448 a.a.), Figure 4 [48]. Two novel *EDARADD*, (c.85G > A, p.E29K) in ED26 and (c.570C > A, p.D190E) mutation in ED27, were identified and predicted to be pathogenic by different in silico algorithms. The structure of 215 a.a. Edaradd remains to be elucidated; nonetheless, the Uniprot entry of Edaradd showed that the (c.570C > A, p.D190E) mutation is located in the death domain (123–202 a.a.) of Edaradd, while (c.85G > A, p.E29K) is located in a region of charged residues (14–29 a.a.), Figure 4 [48]. Many of the *EDARADD* mutations reported to date are missense mutations affecting Edaradd’s death domain, thus, functional consequences of (c.85G > A, p.E29K) would be of interest in terms of the possible role of Edaradd (14–29 a.a.) region on protein structure and function.

## 5. Conclusions

We presented the largest cohort to report on ED in North Africa, expanding the molecular spectrum by presenting seven novel ED mutations that were predicted to target existing and unidentified domains of their corresponding proteins. We recommend the inclusion of *EDA* in targeted sequencing of ED patients, even if XL inheritance is not suggested by pedigree analysis. Our study emphasized the genetic heterogeneity of ED, variable expressivity, and incomplete penetrance. Intrafamilial phenotypic variability was evident, which affects genetic counseling, particularly when prenatal decisions are concerned. Whole exome sequencing would benefit molecularly unidentified cases and provide an insight for future research into potential key player proteins in ED pathogenesis.

## Figures and Tables

**Table 1:** Primers Used for Amplification of Targeted Exons of *EDA*, *EDAR* and *EDARADD* Genes.

Gene-Exon Number	Forward (F) and Reverse (R) Primers	Amplicon Size (bp)	Annealing Temperature (°C)
*EDA*-2	F 5′-TACAGTGGAGGGGAAGATGG-3′	388	61
	R 5′-ACCATGCCCTACCAAGAAGG-3′		
*EDA-4*	F 5′-CTGGGCAACAGAGCAGG-3′	306	60
	R 5′-CCCACTCCTGCTCTCCTAAAG-3′		
*EDA-7*	F 5′-AAAGTTTGGCCTTCTAGGCTAC-3′	418	60
	R 5′-CTTTCAACTCCCTCCCAGTG-3′		
*EDAR-4*	F 5′-CATCTGGAGCCTGAGAGTGG-3′	495	61
	R 5′-GCAGTATCCATGACCCCTGTT-3′		
*EDARADD-2*	F 5′-CTACCTCACCCAGCCAATCC-3′	466	62
	R 5′-CACCTCCAACATGAGCAAAAGA-3′		
*EDARADD-6*	F 5′-CGAGCATTCTGAAATAGTCTTCC-3′	619	60
	R 5′-CTGTTCCACGTCCTTGTCCT-3′		

**Table 2:** Clinical data of molecularly identified ED patients.

Family	Patient	Consanguinity	Sex	Age	Sweating	Hair	Skin	Nails	Degree of Missing Teeth	Peg Shaped Teeth
F1	ED1	−	M	7 y 1 m	H	Sparse	Dry	N	O	+
F2	ED2	−	M	12 y	H	Sparse	Dry	N	O	+
F2	ED3	−	M	7 y	N	Sparse	Dry	N	O	+
F3	ED4	−	M	3 y 5 m	H	Sparse	Dry	N	O	+
F4	ED5	−	F	11 y	H	Sparse	Dry	N	O	+
F4	ED6	−	M	48 y	H	Sparse	Dry	Dysplastic	O	+
F4	ED7	−	F	4 y	N	Sparse	Dry	N	O	+
F4	ED8	−	M	45 y	H	Sparse	Dry	Dysplastic	O	−
F4	ED9	−	F	6 y 7 m	N	Sparse	Dry	N	O	+
F4	ED10	−	M	59 y	H	Sparse	Dry	Dysplastic	O	−
F5	ED11	+	F	9 y	H	Sparse	Dry	N	O	−
F6	ED12	−	M	13 m	H	Sparse	Dry, thin	N	N/A	+
F7	ED13	−	M	1 y 9 m	H	Fair, Sparse	Dry	N	N/A	−
F8	ED14	−	F	3.5 y	H	Sparse	Dry	Dysplastic	N/A	+
F8	ED15	−	F	1.5 y	N	Sparse	Dry	N	N/A	+
F9	ED16	+	M	7 y	H	Sparse	Dry	N	O	+
F10	ED17	+	M	4 y 7 m	H	Sparse	Dry	N	N/A	+
F11	ED18	−	M	2 y 3 m	H	Fair, Sparse	-	N	N/A	−
F12	ED19	+	M	20 y	H	Sparse	Dry	Dysplastic	O	+
F12	ED20	+	M	24 y	H	Sparse	Dry	Dysplastic	O	+
F13	ED21	+	M	11 y	H	Sparse	Dry	N	O	+
F14	ED22	−	M	9 y	H	Sparse	Dry	N	O	+
F15	ED23	−	M	2 y	N	Sparse	-	N	N/A	+
F16	ED24	−	F	10 y	H	Sparse	Slightly dry	N	O	+
F17	ED25	+	M	6 y	H	Sparse	Dry, thin	N	O	+
F18	ED26	+	M	5 y	H	Silky, Sparse	-	N	N/A	+
F19	ED27	+	M	3 y 10 m	H	Sparse	Dry	N	N/A	−
F20	ED28	+	F	3 y	H	Sparse	-	N	N/A	−

Abbreviations: Sweating H: hypohidrotic, N: normal; Nails N: normal; Degree of missing teeth O: oligodontia, H: hypodontia, N/A: not available, mainly for patients younger than 5 years where panoramic radiographs could not be obtained. y: year(s); m: month(s).

**Table 3:** Mutations identified in 20 ED families.

Family	Patient	Gene	Exon (E) or Intron (IVS)	Mutation		Type of Mutation	Genotype	Variant Effect **	Mode of Inheritance ***	Reference
				Nucleotide Change *	Protein Change					
F1–F4	ED1–ED10	*EDA*	E2	c.463C > T	p.R155C	Missense	ED1-4,6,8,10: Hemizygous ED5,7,9: Heterozygous	Pathogenic	XL	[38]
F5–F7	ED11–ED13	*EDA*	E2	c.466C > T	p.R156C	Missense	ED11: Heterozygous ED12,13: Hemizygous	Pathogenic	XL	[38]
F8	ED14 and ED15	*EDA*	E2	c.492delA	p.G165Efs*115	Small deletion	Heterozygous	Pathogenic	XL	**Current study**
F9–10	ED16 and ED17	*EDA*	E4	c.602G > A	p.G201E	Missense	Hemizygous	Pathogenic	XL	**Current study**
F11	ED18	*EDA*	E4	c.620G > A	p.G207E	Missense	Hemizygous	Pathogenic	XL	**Current study**
F12	ED19 and ED20	*EDA*	E4	c.628G > A	p.G210R	Missense	Hemizygous	Pathogenic	XL	**Current study**
F13	ED21	*EDA*	E4	c.659_676delCAGGTCCTCCTGGTCCTC	p.P220_P225del	Small deletion	Hemizygous	Pathogenic	De novo	[39]
F14	ED22	*EDA*	IVS5	c.707-2A > T	p.?	Splicing	Hemizygous	Pathogenic	XL	[40]
F15	ED23	*EDA*	E7	c.865C > T	p.R289C	Missense	Hemizygous	Pathogenic	XL	[41]
F16–17	ED24 and ED25	*EDA*	E7	c.871G > A	p.G291R	Missense	ED24: Heterozygous ED25: Hemizygous	Pathogenic	ED24: De novo ED25: XL	[39]
F18	ED26	*EDARADD*	E2	c.85G > A	p.E29K	Missense	Homozygous	Pathogenic	AR	**Current study**
F19	ED27	*EDARADD*	E6	c.570C > A	p.D190E	Missense	Homozygous	Pathogenic	AR	**Current study**
F20	ED28	*EDAR*	E4	c.204delC	p.Y69Tfs*34	Small deletion	Homozygous	Pathogenic	AR	**Current study**

* Nucleotide changes are based on the following transcripts: *EDA*: **NM_001399.4**, *EDARADD*: **NM_145861.2**, and *EDAR*: **NM_022336.3**; ** Variant effect according to ACMG guidelines [28]; *** Mode of inheritance was confirmed by segregation. XL: X-linked; AR: autosomal recessive; and de novo refers to both parents being wildtype.

**Table 4:** In silico analysis of novel missense mutations.

Gene	Novel Mutation	SIFT	PhD-SNP	Mutation Assessor	Polphen-2	Mutation Taster	PROVEAN	SNPs&GO	CADD Score
*EDA*	c.602G > A (p.G201E)	Not tolerated/Damaging (0.00)	Disease (RI = 2)	Medium (3.2)	Probably damaging (1)	Disease causing (Score = 98)	Deleterious (−4.2)	Disease (RI = 8)	26.3
*EDA*	c.620G > A (p.G207E)	Not tolerated/Damaging (0.00)	Disease (RI = 2)	Medium (3.335)	Probably damaging (1)	Disease causing (Score = 98)	Deleterious (−4.185)	Disease (RI = 1)	26.4
*EDA*	c.628G > A (p.G210R)	Not tolerated/Damaging (0.00)	Disease (RI = 4)	Medium (3.495)	Probably damaging (0.981)	Disease causing (Score = 125)	Deleterious (−2.55)	Disease (RI = 9)	26.0
*EDARADD*	c.85G > A (p.E29K)	Not tolerated/Damaging (0.00)	Disease (RI = 2)	Medium (2.085)	Probably damaging (0.978)	Disease causing (Score = 56)	Neutral (−2.06)	Disease (RI = 8)	28.9
*EDARADD*	c.570C > A (p.D190E)	Not tolerated/Damaging (0.00)	Disease (RI = 4)	Low (1.04)	Probably damaging (0.999)	Disease causing (Score = 45)	Deleterious (−2.97)	Disease (RI = 9)	23.1

Abbreviations: CADD: combined annotation dependent depletion. CADD score ≥ 10 indicates that the variant is predicted to be in the 10% most deleterious substitutions to the human genome, a score of ≥20 indicates the 1% most deleterious, etc. Mutation Assessor predicts functional impact as high (H) or medium (M) or low (L) or neutral (N) at cutoff scores of 3.5, 1.935, and 0.8 between (H&M), (M&L), and (L&N), respectively. MutationTaster scores amino acid changes from 0 to 215 based on evolutionary distance. PhD-SNP: predictor of human deleterious single nucleotide polymorphisms. PolyPhen2 score ranges from 0.0 (benign) to 1.0 (probably damaging). PROVEAN: protein variation effect analyze, score ≤ −2.5 is predicted as damaging; otherwise, it is predicted as neutral. RI: reliability index range from 0 (unreliable) to 10 (reliable), for PhD-SNP and SNPs&GO. SIFT: sort intolerant from tolerant; its threshold for intolerance is 0.05. SNPs&GO: single nucleotide polymorphisms and gene ontology.

## Data Availability

The gene variants presented in this study are openly available in ClinVar database (https://www.ncbi.nlm.nih.gov/clinvar/, accessed on 10 January 2021) under accession numbers (SCV001480041.1- SCV001480048.1, and SCV001762294.1- SCV001762297.1).
